# Defining adaptation within applied physiology – is there room for improvement?

**DOI:** 10.3389/fphys.2024.1459026

**Published:** 2024-08-05

**Authors:** Tadej Debevec, Daniel P. Longman, Jan G. Bourgois

**Affiliations:** ^1^ Faculty of Sport, University of Ljubljana, Ljubljana, Slovenia; ^2^ Department of Automatics, Biocybernetics and Robotics, Jožef Stefan Institute, Ljubljana, Slovenia; ^3^ School of Sport, Exercise and Health Sciences, Loughborough University, Loughborough, United Kingdom; ^4^ Institute of Sport Science (ISSUL), University of Lausanne, Lausanne, Switzerland; ^5^ Department of Movement and Sports Sciences, Faculty of Medicine and Health Sciences, Ghent University, Ghent, Belgium; ^6^ Centre of Sports Medicine, Ghent University Hospital, Ghent, Belgium

**Keywords:** stress, strain, response, adaptation, exercise, environment

## Importance of studying the process of adaptation

There is significant interdisciplinary interest in the biological mechanisms underpinning human adaptation to exercise and environmental stress. For example, improving comprehension of the influence of environmental pressures on human form and function is a primary motivation for much ongoing research within evolutionary anthropology (Longman et al., 2020). In parallel, within applied physiology, an increased understanding of the processes/mechanisms driving adaptation to the strain imposed by exercise and environmental factors could be used to improve health and performance, and to enhance the efficacy of training programs (Longman et al., 2023).

The range of perspectives arising from this widespread interest brings significant opportunities to advance current understanding of adaptive mechanisms via interdisciplinary knowledge-sharing and collaboration. However, realization of this potential requires the consistent and accurate use of key terms relating to the process of adaptation. The interdisciplinary nature of the applied physiology field has led to a highly variable use of terminology and definitions relating to stress, strain and subsequent adaptation. This has introduced a lack of clarity surrounding key terms, hindering scientific progression. The present Opinion aims to illustrate this problem and suggest a more uniform and standardized approach to defining both the processes and the outcomes of adaptation to exercise and environmental pressures.

## Past and current usage of terms describing exercise- and environment-related changes

As evidenced by the recent steady growth of publications on the topic, understanding the influence of exercise and various environmental pressures on human physiology represents one of most important issues within applied physiology. Such knowledge is valuable due to the potential of exercise and environmental pressures to significantly modulate the human body’s form and function as a consequence of homeostatic challenge (Kenney et al., 2022).

Adaptation-related terminology and their definitions often lack consistency in the applied physiology literature. First, humans subjected to stress will develop strain as a result of deviation from homeostasis and it is this strain – rather than the stress itself – that provokes the activation of cellular and molecular signaling pathways that stimulate adaptation, leading to the removal of stress and a return to homeostasis or a new homeostatic level (or maladaptation - the failure of the system to restore homeostasis) (Blum, 2016). Secondly, terms and definitions of adaptation are sometimes taken from evolutionarily contexts and may be inappropriate for use in applied physiology. Specifically, adaptation-related terminology within applied physiology must be considered both from the perspective of form and function and must be applicable within an individual’s lifetime. Mechanisms of adaptation such as natural selection, which occur over many generations, therefore have limited relevance to applied physiology. However, adaptive processes such as phenotypic plasticity, which describes the generation of different phenotypes in response to exercise and different environmental conditions, without the need for genetic change, are applicable (Pigliucci et al., 2006; Wells and Stock, 2007). However, consensus on terms and definitions within applied physiology is often lacking and has been a source of debate over the last century. As outlined in Table 1, while some terms benefit from single definitions in the literature, oftentimes multiple definitions have been applied to the same terms.

**TABLE 1:** Examples of variation in the use of change/adaptation-related terms and definitions used in exercise- and environmental physiology textbooks.

Term	Definition/description	Ref.
Response	Change in body function, reflecting physiological regulation, to every biological event or changing conditions.	Piantadosi (2003)
Acute response	Immediate responses to, and sometimes recovery from, a single exercise bout/exposure aiming to minimize bodily strain.	Kenney et al. (2022)
Adaptive response	The body’s attempts to counteract stressors and reestablish homeostasis. The return to nonstress conditions reflects improved bodily function in the involved organ or system.	Armstrong (2000)
Adaptation	Any response designed to allow an organism or a species to survive represents a form of adaptation. Forms of adaptation are referred to as physiological and genetic adaptations respectively.	Piantadosi (2003)
Adaptation	Trait with a current functional role in the life history of an organism. It is an evolutionary process by which an organism becomes fitted through natural selection for some special activity. Such changes encompass genotypic and phenotypic adaptations as well as behavioral responses to environmental stressors such as heat, cold, and hypoxia.	Gunga (2020)
Adaptation	Changes to the human system induced by exposure to an environmental stressor (also terms, such as: habituation, acclimation, acclimatization). Such changes can encompass both genotypic and phenotypic adaptations as well as behavioral responses to environmental stressors.	Cheung (2010)
Adaptation	Broad umbrella term, encompassing acclimation, acclimatization and habituation, and that can be defined simply as changes to the human system induced by exposure to an environmental stressor. Such changes can encompass both genetic and phenotypic adaptations and can also involve behavioral responses to environmental stressors.	Cheung and Ainslie (2022)
Adaptation	Refers to phenotypic traits that are elicited through natural selection, and it incorporates features of structure, function, or behavior that increase the ability to survive or reproduce in a given environment.	Cheung and Ainslie (2022)
Physiological adaptation	Any functional, structural or molecular change that occurs in an individual because of exposure to change in the environment. It also encompasses genetic responses in the individual, that is, genetic changes in somatic cells that may permanently alter individual fitness (phenotype) but that cannot be passed on to the individual’s progeny.	Piantadosi (2003)
Chronic adaptation	The physiological adaptations that occur with chronic exposure to exercise or training over a period of days and weeks.	Kenney et al. (2022)
Accommodation	Immediate physiological change in the sensitivity of a cell or tissue to change(s) in the external environment.	Armstrong (2000)
Accommodation	Adaptations that occur very quickly following a stress and reverse almost as rapidly when the stress is removed. Accommodation generally describes adaptations that occurs in single cells or tissues.	Piantadosi (2003)
Acclimation/Acclimatization	A complex array of adaptive responses, intermediate in duration, but acclimation is induced experimentally in an artificial environment whereas acclimatization is induced by exposure to natural environments.	Armstrong (2000)
Acclimation/Acclimatization	Acclimation involves physiological adaptation to a single environmental factor or stressor. Acclimatization is the process of adaptation that occurs over a period of days to months in response to a change in natural environment. Acclimatization involves adaptation to two or more environmental factors. Acclimatization results from the interaction or summation of the effects of two or more environmental factors on the response of the body.	Piantadosi (2003)
Acclimation/Acclimatization	Acclimation describes physiological responses to experimentally induced changes, particular, climatic factors such as heat or hypoxia by means of an artificial exposure. Organisms can adjust their morphological, behavioral, physical and/or biochemical traits in response to environmental challenges. Acclimatization, compared to adaptation, occurs within the organism’s lifetime. The process of acclimatization takes a short period of time (days to weeks). The individual organism adjusts gradually during this time span to the natural environment, allowing it to maintain performance across a range of environmental conditions.	Gunga (2020)
Acclimation/Acclimatization	Acclimation involves adaptive physiological responses to experimentally induced changes in particular climatic factors. Acclimatization refers to adaptive physiological or behavioral changes occurring in an individual in response to changes in the natural climate.	Cheung and Ainslie (2022)
De-acclimation/De-acclimatization	Any process that completely or partially reverses an adaptive response is known as de-acclimation or de-acclimatization.	Piantadosi (2003)
Habituation	A dampening of the normal response to a stressor.	Armstrong (2000)
Habituation	Decrease in response to a constant stimulus or a decrease in intensity over time of one or more responses to a stimulus of constant intensity.	Piantadosi (2003)
Habituation	Habituation (also termed sensitization) refers to a reduction in the perception of a repeated stimulation.	Cheung and Ainslie (2022)
Cross-acclimation	Influence of earlier adaptation to one stressor on subsequent adaptation to a new environment that may or may not contain the initial stressor. This can be positive or negative.	Piantadosi (2003)
Cross-adaptation Cross-acclimation Cross-acclimatization Cross-tolerance	Generic term to describe two distinct components: cross-acclimation/cross-acclimatization and cross-tolerance. Adjustments derived from exposure to a simulated environment. Adjustments derived from exposure to a natural environment. Cellular and molecular pathways to adaptations between thermal and hypoxic environments.	Cheung and Ainslie (2022)
^a^Cross adaptation ^a^Crossed sensitization or negative cross-adaptation, ^a^Crossed resistance or positive cross-adaptation.	Long-term exposure (either continuous or intermittent) to a given adverse environment not only leads to an increase in tolerance to that environment but leads also to gains or losses in tolerance to other adverse factors, ones which the adapted organism had never encountered previously. For any given adaptation, some side effects may be either good or bad. High degrees of physiologic displacements resulting from exposure to a test environment are taken as evidence of cross sensitization or negative cross adaptation, while low degrees of displacement under such conditions denote crossed resistance or positive cross-adaptation.	Hale (1969)
^a^Cross-adaptive effect ^a^Interstress adaptation	A generic autonomic adaptation provided by a specific environmental stress (e.g., repeated cold air exposures) can result in a reduction in the sympathetic response to novel environmental stimuli (e.g., hypoxia).	Leblanc (1969)
^a^Cross stress adaptation ^a^Cross sensitization	Physiological and biochemical phenomenon in which there is a reduced stress responsiveness to a novel stressor in previously stress adapted organisms. Due to the bidirectional trend of changes, the adaptive changes caused by one stressor may make the organism more fit to resist the adverse effects of another type of stressor. Phenomenon characterized by an exaggerated response to a novel stressor following exposure to repeated chronic stress.	Chauhan et al. (2015)
^a^Cross acclimation ^a^Cross tolerance	Adaptation to one environmental stressor can induce protective responses upon exposure to other stressors if they share common adaptive responses. This phenomenon is termed cross-acclimation, when physiological strain is attenuated or cross-tolerance, when improved cellular protection is observed.	Lee et al. (2016)
^a^Cross-adaptation ^a^Cross-acclimation ^a^Cross-acclimatization ^a^Cross-tolerance ^a^Thermotolerance ^a^Acquired cellular thermotolerance	Generic term used to describe the phenomenon whereby alternative environmental interventions, e.g., heat acclimation (HA) or cold acclimation (CA), may be a beneficial alternative to altitude interventions, providing physiological stress and inducing adaptations observable at altitude. Cross-acclimation (adaptation derived from a simulated environment) or cross-acclimatization (adaptation from a natural environment) are adopted to define specific adaptations made at a physiological level due to commonality with the typical descriptions of the acquired HA or CA phenotype. Cross tolerance is the adopted nomenclature appropriate to describe cellular and molecular pathways relevant to adaptations between the thermal and hypoxic environments due to common pathways with thermotolerance (also known as acquired cellular thermotolerance.	Gibson et al. (2017)
^a^Heat acclimation-mediated cross-tolerance (HACT)	The primary outcome of heat acclimation is increased thermotolerance by enhancement of innate cytoprotective pathways. These pathways produce “ON CALL” molecules that can combat stressors to which the body has never been exposed via cross-tolerance mechanisms.	Horowitz (2017)

^a^ Depicts terms and definitions used in select peer reviewed publications.

For example, there is significant variation in how the term adaptation is defined. While Piantadosi (2003) highlights the need to differentiate between physiological and genetic forms of adaptation, Cheung and Ainslie (2022) use the term adaptation as a broad umbrella term encompassing acclimation, acclimatization and habituation and resulting in both genetic and phenotypic adaptations as well as behavioural responses to environmental stressors. Similarly, acclimation is often defined as physiological adaptation to a single environmental factor or stressor, regardless of the mode of environmental pressure (Piantadosi, 2003). However, numerous authors have also argued that the term should be solely employed to describe an array of adaptive responses induced experimentally (e.g., using an artificial environment) (Armstrong, 2000; Gunga, 2020; Cheung and Ainslie, 2022).

Precisely defining the process of adaptation and subsequent outcomes becomes even more challenging and important when addressing multi-stressor scenarios. Indeed, the outcomes of different combinations of environmental stressors (e.g., heat, cold and hypoxia) and exercise levels (e.g., modality and load) are often assessed in terms of their potential to improve performance in the targeted environmental condition (e.g., exercise in the heat to improve performance in the heat) or in a different condition (e.g., exercise in the heat to improve performance in hypoxic condition) (Gibson et al., 2017; Sotiridis et al., 2022).

Similarly, there is significant interest in the independent and combined effects of various exercise types (e.g., endurance vs. strength training) and intensities (various domains) on performance in the targeted exercise types and intensities as well as in the “transfer” of effects to other domains. This is further complicated by the complexities associated with delineating the multi-stressor effects of exercise and environment and the potential differential adaptations when employing the stressors in a concomitant (e.g., endurance and strength training during the same period) or sequential (e.g., training in the heat following an altitude training camp) application. Unfortunately, as also shown in Table 1, inconsistency in the use of terminology has led to ambiguity of meaning. Specifically, various terms (such as cross-tolerance, cross-adaptation, cross-acclimatization, heat acclimation-mediated cross-tolerance and interstress adaptation) are often used interchangeably and, crucially, are not always clearly defined. This leads to a lack of clarity regarding the employed duration (acute vs. chronic) and mode (natural vs. artificial) of the environmental pressures as well as the nature of the subsequent changes (e.g., molecular, cellular, physiological and/or functional).

## Evolutionary insights

The process of adaptation to environmental pressures is a core tenet of evolutionary theory. As a result, there has been increased interest in recent years in applying conceptual frameworks used by evolutionary anthropologists to the field of applied physiology (Shirley et al., 2022; Longman et al., 2023), and vice versa (Longman et al., 2020).

Within evolutionary biology, adaptation describes the process by which an individual adjusts to their environment to maximize their evolutionary fitness (that is, their ability to survive and reproduce). The principal genetic mechanisms of adaptation include a) natural selection – the process by which individuals with traits that are better suited to their environment tend to survive and reproduce more successfully than those lacking such traits, leading to the gradual increase in the frequency of genes encoding those advantageous traits within a population over generations (Darwin, 1859); b) genetic drift – the change in frequency of alleles within a population over successive generations due to random chance (Lynch et al., 2016); c) gene flow – the movement of genes or alleles between populations of the same species, typically through the migration of individuals or the transfer of gametes (Lenormand, 2002); and d) epigenetic inheritance – the transmission of chemical modifications to DNA or associated proteins from one generation to the next, influencing patterns of gene expressions and potentially affecting traits without changes in the DNA sequence itself (Stajic and Jansen, 2021).

Faster-acting adaptive mechanisms, active within an individual’s lifetime, include a) developmental plasticity – the generation of different morphological, physiological and behavioral phenotypes in response to variation in environmental conditions experienced during key stages of development (Nettle and Bateson, 2015); b) phenotypic plasticity – the ability of an individual’s genome to produce different phenotypes in response to exposure to varying environmental cues, at any life stage (Pigliucci et al., 2006); and c) behavioral adaptation – the process of altering behavior in response to a changing environment (Dunbar, 2020). The adaptative features resulting from the above processes may be structural (e.g., morphological features), physiological (including metabolic changes) or behavioral (including changes in how an individual interacts with their environment) in nature. Of these, phenotypic plasticity is perhaps most relevant to applied physiology as it facilitates adaptation at a timescale that is relevant for athletic training.

## Proposed adaptation-related terminology for use within applied physiology

Precise definitions of adaptation-related terminology are therefore necessary for use within applied physiology to ensure a clear understanding of experimental/intervention design, the associated results and the mechanisms underlying the observed effects. We therefore propose the following definitions to simplify and standardize terminology within the applied physiology field:

• ‘Response’ to describe acute changes to exercise and/or exposure to environmental factors;
• ‘Adaptation’ to describe subacute and chronic changes induced by exercise intervention(s);
• ‘Acclimation’ to describe subacute and chronic changes induced by exposure to environmental factors using artificial/simulated environmental stress;
• ‘Acclimatization’ to describe subacute and chronic changes induced by natural environmental stress;
• ‘Habituation’ to describe a reduction in the perception of a repeated stimulus which is not necessarily associated with actual molecular, cellular, physiological or functional change;
• ‘Cross-adaptation’ to describe subacute and chronic changes induced by one exercise intervention type that also influence (subacute and chronic) changes induced by another exercise intervention;
• ‘Cross-acclimation’ to describe subacute and chronic changes induced by exposure to one environmental factor - using the artificial/simulated environmental stress - that also influence (subacute and chronic) changes induced by another environmental stressor;
• ‘Cross-acclimatization’ Cross-acclimatization to describe subacute and chronic changes induced by one environmental factor - using the actual/natural environmental stress - that also influence (subacute and chronic) changes induced by another environmental stressor.

We further suggest that, in addition to increasing clarity surrounding the use of the key terms defined above, it is also crucial to precisely define:

• The exact intervention/process that was employed to provoke the observed changes;
• The method of applying multiple stressors scenarios (i.e., independent, sequential and/or combined at the same time);
• Whether the observed structural and functional changes are molecular, cellular, physiological and/or functional.

While we appreciate that this proposed terminology may not comprehensively address all terms requiring clarification, we hope that this Opinion will a) facilitate a much-needed discussion leading to a consensus on the correct way to discuss stress, strain and adaptation within the applied physiology field, and b) decrease ambiguity and thereby optimize scientific progress and understanding.
